# Understanding Fascial Tissue on the Molecular Level—How Its Unique Properties Enable Adaptation or Dysfunction

**DOI:** 10.3390/ijms27010160

**Published:** 2025-12-23

**Authors:** Karen B. Kirkness, Suzanne Scarlata

**Affiliations:** 1 Health Professions Education Unit, Hull York Medical School, York YO10 5DD, UK; 2 Department of Chemistry and Biochemistry, Worcester Polytechnic Institute, Worcester, MA 01609, USA; sfscarlata@wpi.edu

**Keywords:** calcium signaling, *HAS2*, hyaluronic acid, fascia, mechanotransduction, extracellular matrix, tissue adaptation, morphogenetic field, CD44, fasciacytes

## Abstract

Despite extensive research on fascial mechanobiology, no unified mechanotransduction framework has been established to explain how mechanical forces translate into adaptive cellular responses in fascial tissue. This narrative review synthesizes evidence from mesenchymal cell and fibroblast research to propose the Ca^2+^–Hyaluronan (CHA) axis as a comprehensive mechanotransduction feedback loop for fascia phenomenology. The CHA framework describes how mechanical stress activates Ca^2+^ channels (Piezo1, TRPV4, P2Y2), triggering *HAS2*-mediated hyaluronan (HA) synthesis. The molecular weight of synthesized HA then determines receptor signaling outcomes: high-molecular-weight HA binds CD44 to promote tissue stability and quiescence, while low-molecular-weight HA fragments activate RHAMM to drive remodeling and repair—a dynamic oscillation termed “Quiet or Riot.” Three key conclusions emerge: First, the CHA framework is well supported by existing literature on mesenchymal cells, providing a testable model for fascial mechanobiology. Second, HA molecular weight dynamics and CD44/RHAMM oscillation have direct implications for optimizing movement, manual therapy, and rehabilitative interventions. Third, while HA-CD44/RHAMM signaling is broadly implicated in tissue remodeling, Ca^2+^-dependent regulatory mechanisms specific to fasciacytes require experimental validation. A critical translational gap remains: the absence of quantitative mechanical thresholds distinguishing beneficial from pathological loading limits clinical application. Future research should employ 3D matrix models, live imaging, receptor manipulation, and omics profiling to establish these thresholds and validate the CHA framework in fasciacytes. Understanding fascial mechanotransduction through the CHA loop may transform approaches to movement prescription, manual therapy, and treatment of fascial dysfunction.

## 1. Introduction

### 1.1. HA in Fascia: Structure, Function, and Cellular Sources

Recent literature recognizes fascia as a globally distributed, highly specialized connective tissue with both fibrous and gel-like hyaluronic acid (HA) components, reflecting its diverse anatomical and functional roles [1,2,3]. HA is a key functional component of the extracellular matrix (ECM) that supports cellular structure. It is an evolutionarily ancient and highly conserved polysaccharide found across a wide range of organisms, from bacteria to vertebrates, underscoring its fundamental biological importance [4,5].

**Figure 1:**
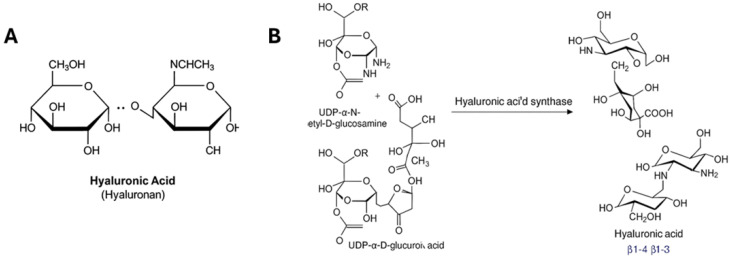
Chemical properties of hyaluronic acid (HA). (**A**) structure of HA showing that it is a linear polysaccharide composed of repeating disaccharide units of β-1,4-D-glucuronic acid and β-1,3-N-acetyl-D-glucosamine. (**B**) Chemical reaction showing the catalysis by HAS involves the polymerization of hyaluronic acid (HA) from two sugar nucleotide substrates, UDP-N-acetylglucosamine and UDP-glucuronic acid.

HA’s unique physicochemical properties—including its capacity to bind up to 1000 times its weight in water, its viscoelastic behavior, and its role in cell signaling—make it essential for maintaining tissue hydration and modulating cellular responses to mechanical and biochemical stimuli [6,7,8]. HA is abundant in the loose connective tissue of fascia, facilitating gliding in fascial planes, and supporting viscoelasticity and tissue adaptability [9,10,11]. The amount, weight, and properties of HA in fascia vary by anatomical site, correlating with the degree of required tissue gliding and mechanical function [9,12]. See Figure 1.

Specialized cells called fasciacytes—recently identified as distinct from fibroblasts—are dedicated to producing HA-rich ECM, with high expression of the *HAS2* gene (see below), which synthesizes high molecular weight HA crucial for hydration and homeostasis [3,13,14]. The amount and properties of HA in fascia vary by anatomical site, correlating with the degree of required tissue gliding and mechanical function.

### 1.2. HAS2: The Key Contributor of Normal HA in the ECM

**Figure 2:**
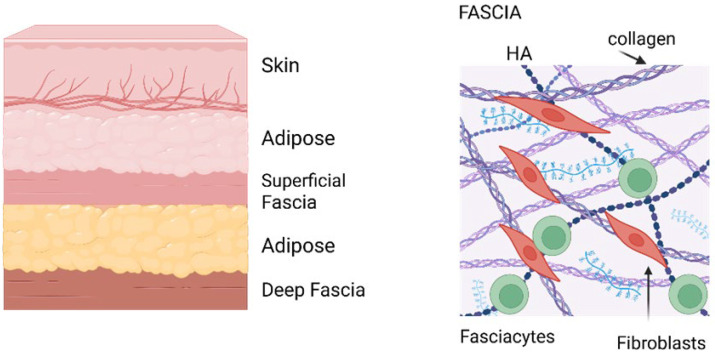
Anatomy of fascia. (**Left**)—Cartoon of layers above muscles showing the location of fascia. (**Right**)—Fascia organization. Image created with BioRender.com, accessed on 11 November 2025.

The synthesis of HA is catalyzed by three hyaluronan synthase isoforms (*HAS1*, *HAS2*, and *HAS3*), among which *HAS2* demonstrates unique regulatory properties and tissue-specific expression patterns. *HAS2* is the key isoform responsible for producing high molecular weight HA essential ECM homeostasis. All mesenchymal cells express *HAS1/2/3*, but *HAS2* concentration is by far the highest and linked to normal ECM function. Research on *HAS2* is most extensive in fibroblasts and mesenchymal stem cells (MSCs), reflecting their central roles in tissue repair, ECM production, and immunomodulation [15,16]. See Figure 2.

### 1.3. HAS2-Expressing Cells: From Fibroblasts to Fasciacytes

The grouping of fibroblasts and mesenchymal stem cells (MSCs) as primary research models is scientifically justified given their striking similarity. These cell types are often indistinguishable by standard laboratory criteria, sharing morphology, surface markers, gene expression, and differentiation potential, as both can differentiate into adipocytes, chondrocytes, and osteoblasts, and both exhibit immunomodulatory properties [17,18,19,20,21]. Some researchers propose that fibroblasts may represent a differentiated or aged form of MSCs, or that MSCs are a specialized subset of fibroblasts, further supporting the validity of using findings from one cell type to inform understanding of the other.

Fasciacytes are a distinct cell type within fascia characterized by the highest expression of *HAS2*, which differentiates them functionally from classical fibroblasts and MSCs [22]. While fasciacytes share some markers with fibroblasts, such as vimentin positivity, they exhibit unique morphological features and express markers like S-100A4, suggesting a specialized role in producing the HA-rich ECM critical for fascial gliding. Fibroblasts and MSCs also produce HA via *HAS2*, which regulates processes like cell migration, senescence, and fibrosis, but their *HAS2* expression and HA signaling roles vary by tissue context and pathological state [23,24].

### 1.4. Fascia Mechanotransduction: Multiple Pathways

Fibroblasts have been shown to orchestrate fascia’s structural integrity, mechanical responsiveness, and repair processes through HA extrusion, ECM synthesis, contractility, and interaction with immune signals [12,25,26]. Fascial tissues rapidly adapt to mechanical demands through such autonomous, cell-intrinsic mechanotransduction mechanisms. Key mechanosensors in fascia that have long dominated the literature include focal adhesions (FAs), integrin-associated complexes that link the extracellular matrix (ECM) to the cytoskeleton and transduce mechanical signals into biochemical responses, regulating cell adhesion, cytoskeletal remodeling, and gene expression [27,28,29].

### 1.5. YAP as a Downstream Mechanotransducer

Recent evidence highlights Yes-Associated Protein (YAP) as a key mechanotransducer in deep fascia fibroblasts, where mechanical stimulation activates YAP signaling to promote ECM remodeling and fibrogenesis [30,31]. In thoracolumbar fascia fibroblasts, mechanical stimuli increase active YAP, upregulating collagen and hyaluronan-binding protein (HABP2) expression, creating a feed-forward loop where YAP-driven ECM deposition increases matrix stiffness, further activating mechanosensitive pathways [32]. This positions YAP as a central downstream effector translating mechanical stimuli into profibrotic gene expression, linking tissue homeostasis and pathological states such as fibrosis and myofascial pain [33,34,35].

### 1.6. HA: The Global Mechanotransductive Gel

HA molecular weight modulates cellular signaling across multiple cell types [36,37,38] where high molecular weight (HMW) HA increases tissue stiffness while fragmentation produces low molecular weight (LMW) HA that promotes inflammation [11,39]. However, direct evidence for differential YAP responses to HMW versus LMW HA specifically in fascia fibroblasts remains limited. Researchers have explicitly acknowledged this gap and the “widespread fog” regarding HA molecular weight effects on specific biological processes, particularly YAP/TAZ signaling in tissue-specific contexts [36,40,41].

### 1.7. Aims and Scope of This Review

HA is predominantly viewed as a passive gel substrate for tissue hydration and lubrication. Here, we argue that HA is an active participant in mechanotransduction. Despite extensive evidence of HA molecular weight’s influence on cellular adaptation and the critical importance of Ca^2+^-mediated mechanotransduction in fascial function, no comprehensive biochemical feedback loop has been proposed. We aim to demonstrate that the evidence integrating mechanical stress, calcium signaling, HA synthesis, and molecular-weight-dependent receptor signaling has been clearly established in cell types relevant to fascia research. This review argues that the Ca^2+^-HA (CHA) axis provides the missing unified framework. CHA identifies the molecular pathway in which HA functions not merely as a structural matrix but also as a central signaling molecule whose molecular weight orchestrates oscillations between tissue adaptation and homeostasis. The scope of this framework is supported by abundant research on mesenchymal cells, positioning HA as integral to fascial mechanobiology. It offers a testable model for understanding tissue responses to mechanical loading, relevant to clinicians, movement educators, and manual therapists.

## 2. The Calcium-HA (CHA) Axis: Overview

### 2.1. The CHA Axis: The Mechanotransduction Feedback Loop in Fascia

As detailed below, HA synthesis is regulated by intracellular Ca^2+^ levels that are mobilized by several mechanical cues. We propose that the tight coupling between Ca^2+^-dependent pathways and *HAS1/2/3* expression represents a fundamental principle that enables fascia to function as a responsive, adaptive tissue interface that operates largely independently of higher-level neural or endocrine control. This hypothesis is based on evidence from multiple research domains, including mechanobiology, cell signaling, tissue engineering, and developmental biology.

### 2.2. The Fundamental Role of Calcium in Cell Signaling

Calcium ions (Ca^2+^) function as one of biology’s most versatile and ubiquitous second messengers, capable of translating diverse environmental stimuli into rapid, coordinated cellular responses [42]. The use of Ca^2+^ as a signaling molecule exploits several unique properties. Its low resting intracellular concentration (typically ~100 nM) compared to extracellular levels (~1–2 mM) creates a steep electrochemical gradient. Its ability to bind rapidly and reversibly to numerous proteins, altering their conformation and activity, enables cellular responses. The existence of highly conserved cellular machinery has evolved across the kingdoms of life to generate, propagate, and terminate Ca^2+^ signals with precise spatiotemporal control [43].

In the context of tissue adaptation and ECM remodeling, Ca^2+^ signaling serves as an integration point for multiple stimulus modalities. Mechanical forces, detected by mechanosensitive ion channels and integrin-mediated adhesions, can trigger Ca^2+^ influx directly [44]. Chemical signals, including ATP released from stressed or damaged cells, activate G-protein coupled receptors that mobilize intracellular Ca^2+^ stores [45].

### 2.3. Calcium-Driven HA Synthesis as Mechanical Adaptation

Elevated Ca^2+^ activates the *HAS2* signaling cascade leading to increased HA synthesis within hours. Because of its extraordinary water-binding capacity, this newly synthesized HA increases tissue hydration. This hydration distributes mechanical loads more evenly, protecting against compressive damage [6], contributing to the viscosity buffering properties of the fluid between fascial planes, easing movement. HA influences cell adhesion, migration, and signaling through its interactions with CD44, RHAMM, and other receptors, facilitating tissue remodeling and repair [46].

Thus, the *HAS2* pathway represents a form of real-time mechanical adaptation. We argue that fascia “feels” the increased mechanical demand and immediately increases production of its primary hydrating molecule. The system operates as a self-lubricating bearing: when mechanical demand increases, the system automatically produces more lubricant.

## 3. Mechanotransduction and Calcium Signaling

### 3.1. Mechanical Forces as Primary Fascial Stimuli

Mechanical forces represent perhaps the most constant and physiologically relevant stimulus for fascial tissues. Every movement, posture change, and load-bearing activity generates mechanical strains that must be sensed and responded to maintain tissue integrity and function. Cells detect mechanical forces through multiple mechanisms, including stretch-activated Ca^2+^ channels, integrin-mediated adhesions that link the cytoskeleton to the ECM, and primary cilia that act as mechanical antennae [44,47].

**Figure 3:**
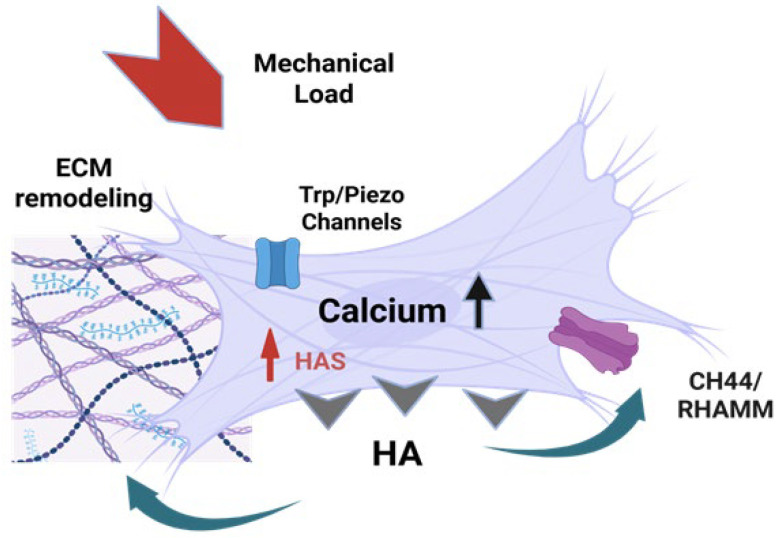
The CHA feedback loop in Fascia. Mechanical forces (stretch, pressure, shear stress) deform mesenchymal cells, initiating a biochemical feedback loop that links mechanical stimuli to hyaluronan synthesis and cellular adaptation. Mechanosensitive calcium channels (Piezo1/2, TRPV4) are activated by mechanical deformation [12,55,56,57,58], leading to rapid Ca^2+^ influx and elevated intracellular calcium levels [45,57,59], which then sets off signaling cascades that lead to the upregulation of *HAS1/2/3* and HA synthesis [45,55,57,60]. Newly synthesized HA is extruded into the ECM, where it increases ECM hydration and is sensed by CD44 and RHAMM receptors on the cell surface, which modulate further HAS expression and cellular responses, creating a self-regulating feedback loop [12,55,57]. Image created with BioRender.com, accessed on 11 November 2025.

Many mechanosensing mechanisms directly or indirectly modulate intracellular Ca^2+^ levels, establishing a critical link between mechanical stimulation and cellular biochemical responses. Mechanically gated (stretch-sensitive) Ca^2+^ channels embedded in the plasma membrane allow Ca^2+^ influx in direct proportion to membrane deformation, essentially converting mechanical force into an ionic signal. The magnitude and duration of Ca^2+^ entry through these channels depend on both the intensity and persistence of the applied mechanical stress as shown in Figure 3 and described in Table 1.

### 3.2. Dose–Response: Intensity and Duration of Mechanical Stress

Higher levels of mechanical stress (e.g., greater shear force or stretch amplitude) result in larger and more sustained increases in intracellular Ca^2+^. This dose-dependent Ca^2+^ response directly translates to proportional *HAS2* activation [53], as intense stress generates high Ca^2+^ signals that activate signaling molecules which allow cellular memory and greater *HAS2* transcription. Tissues experiencing higher mechanical loads thus produce more HA, exactly when and where it is needed most [54].

The duration of mechanical stress application also shapes the Ca^2+^ response: short, intense pulses trigger transient Ca^2+^ spikes, while sustained or repetitive stress leads to prolonged or cumulative Ca^2+^ elevations [48,49]. The temporal pattern of Ca^2+^ signaling determines how long *HAS2* remains upregulated. Transient Ca^2+^ spikes from brief stress trigger short bursts of *HAS2* expression that self-terminate through negative feedback (ATP → adenosine) [34,52,53,54], whereas sustained or repetitive stress maintains elevated Ca^2+^ longer, prolonging *HAS2* activation and producing more sustained HA synthesis. This temporal sensitivity prevents the system from wasting resources responding to trivial, momentary perturbations while mounting robust responses to persistent mechanical challenges.

### 3.3. Mechanosensitive Channel Diversity

Three primary mechanosensitive Ca^2+^ channels—Piezo1, TRPV4, and TRPC5—work in concert to provide fascial cells with a nuanced capacity to detect diverse mechanical stimuli (Table 2). Cells can express unique combinations of mechanosensitive channels, allowing them to be “tuned” to the dominant mechanical forces in their microenvironment—some regions more responsive to shear (Piezo1/TRPV4) and others to stretch or compression (Piezo1/TRPC5). This channel diversity enables different fascial regions to calibrate their *HAS2* response, resulting in spatially appropriate HA synthesis and lubrication tailored to local mechanical demands.

## 4. The *HAS2*–Calcium Signaling Cascade: Molecular Mechanisms

### 4.1. From Mechanical Force to HAS2 Transcription

Mechanical forces initiate a multi-step cascade that translates physical stimuli into *HAS2* gene activation. First, mechanosensitive Ca^2+^ channels detect tissue deformation and generate initial Ca^2+^ influx. This primary signal is then amplified through a secondary mechanism: when cells sense mechanical force through their structural connections to the surrounding matrix, integrins cluster at focal adhesion sites and activate focal adhesion kinase (FAK), Src family kinases, and phospholipase C [47]. These molecules trigger Ca^2+^ release from internal storage compartments (the endoplasmic reticulum) through IP_3_ receptor activation, providing a secondary, amplified Ca^2+^ wave that complements the initial influx from stretch-activated channels [65].

This combined Ca^2+^ signal must then be translated into genetic instructions that activate *HAS2* synthesis. The pathway from Ca^2+^ elevation to *HAS2* gene expression involves multiple signaling intermediates and transcription factors—including CaMKII, PKC, MAPK pathways, and CREB—that work sequentially to amplify the signal while providing multiple points for regulation and integration with other pathways (detailed in Section 4.2). Studies in keratinocytes have provided the most detailed characterization of this cascade [45], though evidence shows it is a broadly conserved mechanism found in neurons, epithelial, and other cell types [66,67]. Mechanical load changes trigger Ca^2+^ elevation, which drives *HAS2* upregulation, leading to increased HA synthesis and enhanced tissue hydration and gliding capacity. This coupling allows for continued movement, which closes the loop.

### 4.2. The Molecular Relay: CaMKII, PKC, MAPK, and CRE

#### 4.2.1. Signal Amplification: The Molecular Megaphone

Signal amplification in cellular signaling is a process of molecular multiplication, in which each kinase acts as a molecular megaphone, activating multiple downstream targets simultaneously. Ca^2+^ signaling integrates with other pathways involving YES kinase and hyaluronan (HA) receptors such as CD44 and RHAMM to modulate *HAS2* expression and extracellular matrix remodeling. These interactions provide additional regulatory layers beyond Ca^2+^-dependent kinases and transcription factors, allowing cross-talk that fine-tunes *HAS2* gene activation and cellular responses [68]. Growth factors like FGF9 further promote *HAS2* expression through activation of the Wnt/β-catenin/TCF7L2 pathway, a critical signaling axis involved in development, tissue homeostasis, and disease [69,70].

The Wnt/β-catenin pathway regulates gene transcription by stabilizing β-catenin, which partners with TCF/LEF transcription factors. The pathway drives target gene expression, including *HAS2*, linking extracellular signals to genetic programs [68,69]. Convergence of Ca^2+^ signaling with YES kinase, HA receptor pathways, and Wnt/β-catenin signaling highlights a complex network where multiple inputs integrate to control *HAS2* expression and extracellular matrix dynamics. Such multi-pathway integration ensures precise regulation of *HAS2* in response to diverse physiological and pathological stimuli.

This cascading mechanism allows a small initial signal—in this case, a brief Ca^2+^ influx—to generate a robust cellular response by progressively increasing its strength and range. The multi-step pathway not only amplifies the signal but also provides critical regulatory checkpoints, enabling the cell to fine-tune its response to different magnitudes and durations of mechanical stimulation, ultimately translating mechanical force into precise genetic activation.

#### 4.2.2. The Sequential Relay: CaMKII → PKC → MAPK → CREB

The first major responder is Ca^2+^/calmodulin-dependent protein kinase II (CaMKII). This serine/threonine kinase is directly activated when Ca^2+^ binds to calmodulin, thereby switching on CaMKII’s enzymatic activity. Even after the Ca^2+^ signal fades and returns to baseline levels, CaMKII can remain active for minutes or even hours, allowing brief Ca^2+^ spikes to generate lasting cellular changes [37,67] (Table 3). Simultaneously, multiple isoforms of protein kinase C (PKC) become activated in response to both elevated Ca^2+^ and diacylglycerol (DAG), which is produced alongside IP_3_ when phospholipase C splits membrane lipids, and once activated, PKC begins phosphorylating components of the MAPK cascade, creating a critical connection that links the initial Ca^2+^ signal to a major cellular communication highway [37].

The mitogen-activated protein kinase (MAPK) pathways—particularly ERK1/2, p38, and JNK—act as the middle segment of this relay, passing the signal along by phosphorylating transcription factors that can enter the cell nucleus and bind directly to DNA, with ERK1/2 showing particularly strong involvement in activating *HAS2* expression [37]. The final critical player is CREB (cAMP response element-binding protein), a transcription factor that receives phosphate modifications from multiple kinases, including both the MAPK cascade and CaMKII. Once phosphorylated and activated, this “master switch” binds to specific DNA sequences called cAMP response elements (CREs) in the *HAS2* gene’s promoter region, recruiting additional coactivators that work together to initiate transcription and produce messenger RNA that will ultimately be translated into *HAS2* enzyme protein [37].

#### 4.2.3. Temporal Dynamics of the Cascade

The cascade unfolds according to a precise temporal schedule: initial Ca^2+^ elevation occurs within seconds of stimulus application, activation of intermediate kinases follows within minutes, *HAS2* mRNA levels begin to increase within 1–2 h and peak at 4–6 h post-stimulation, and newly synthesized HA accumulates in the pericellular and extracellular space over the subsequent hours, with maximal accumulation typically observed 6–12 h after the initial stimulus [37]. We argue that this represents a temporal cascade allowing fascia to detect mechanical challenges almost instantly while mounting a sustained adaptive response over hours. This bridges the critical gap between immediate sensing and meaningful tissue-level change.

## 5. HA Extrusion: Simultaneous Synthesis and Secretion

### 5.1. HAS2: Both a Synthase and a Translocator

*HAS2* functions like a biological 3D printer, building the HA polymer chain on one side of the cell membrane while simultaneously threading it through to the other side, manufacturing and delivering the product in a single continuous process. As a Class I membrane-integrated enzyme, *HAS2* uses a processive chain elongation mechanism, catalyzing the alternating addition of UDP-activated sugars (UDP-glucuronic acid and UDP-*N*-acetylglucosamine) to the growing HA chain while channeling the polymer through a transmembrane pore directly into the extracellular space [75].

This integration of a cytosolic catalytic domain with a channel-forming transmembrane region allows *HAS2* to couple HA polymerization with secretion efficiently. Post-translational modifications such as O-GlcNAcylation stabilize *HAS2* in the membrane and enhance HA production, whereas phosphorylation by AMPK can inhibit HA secretion, linking HA extrusion to cellular energy status [76]. Increased *HAS2* expression or activity leads to elevated HA synthesis and extrusion, which can promote pathological remodeling processes, such as in pulmonary hypertension and cancer [77,78,79]. Thus, *HAS2* functions as both a synthase and a translocator, producing and extruding HA directly into the ECM through its membrane-embedded structure.

### 5.2. Negative Feedback and Signal Termination

#### Mechanisms for Termination

The elegant responsiveness of the Ca^2+^-HA loop and its ability to detect mechanical stress and rapidly mobilize adaptive responses through the calmodulin–CaMKII cascade carries an inherent danger. Any signaling system capable of rapid, amplified responses must also possess mechanisms for termination, or it risks runaway activation that could damage rather than protect tissue. Uncontrolled Ca^2+^ signaling leads to excitotoxicity, cellular dysfunction, and ultimately cell death. Specifically, unchecked *HAS2* activation and excessive HA synthesis can create pathological tissue states (see Table 4). HA’s strong hydrophilic nature means that excessive production leads to pathological water retention and tissue edema, particularly in inflamed or injured tissues [71,72]. Conversely, dense, overly viscous HA-rich matrices impede normal cellular migration, affecting tissue repair and immune responses—the very processes that appropriate HA synthesis should facilitate [72,73,74,80].

Perhaps most concerning, excessive *HAS2*-driven HA synthesis promotes progressive fibrosis through activation of fibroblasts and myofibroblasts, leading to pathological ECM deposition and tissue scarring in liver, kidney, and lung tissues [73,74,79,81]. In pathological contexts such as cancer, high *HAS2*/HA levels create a fibrotic, immunosuppressive microenvironment that supports tumor progression and therapeutic resistance [73,77,80,82].

## 6. HA–Receptor Signaling

### 6.1. CD and RHAMM Oscillation Powers the CHA Feedback Loop

**Figure 4:**
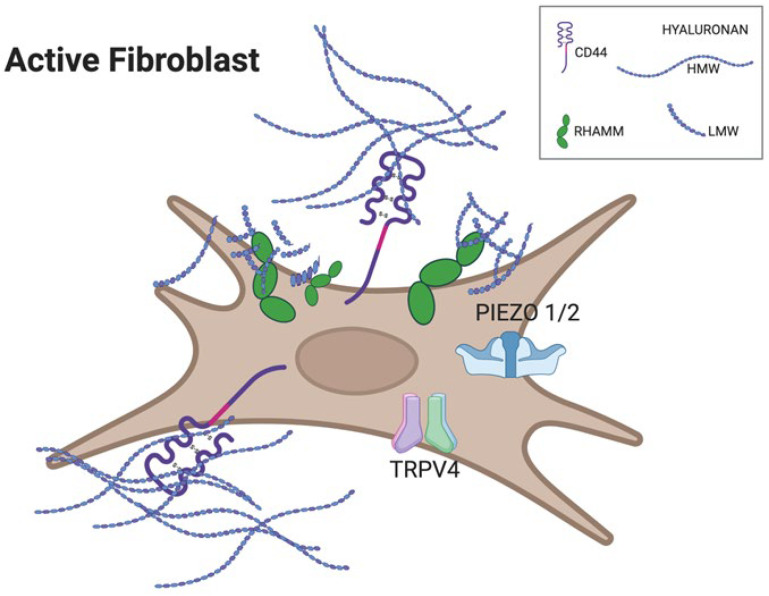
Cartoon of an active fibroblast illustrating differential receptor engagement by hyaluronan fragments. Long HMW-HA chains preferentially bind and cluster CD44 at the cell surface, promoting structural integrity and anti-inflammatory signaling. Short LMW-HA fragments accumulate around RHAMM and stimulate mechanosensitive ion channels TRPV4 and PIEZO1/2, driving Ca^2+^ entry and initiating YAP-dependent transcriptional responses linked to fibrosis. Created with BioRender.com, accessed on 11 November 2025.

Once extruded, the HA accumulates in the pericellular matrix and binds to its primary receptors, CD44 and RHAMM (Receptor for Hyaluronan-Mediated Motility), on the cell surface (see Figure 4). CD44 and RHAMM are mechanotransducers that convert HA binding into ion flux, cytoskeletal change, and gene expression. CD44 engages with high-molecular-weight HA (HMW-HA), which triggers multiple signaling pathways, including those involving Src family kinases, Rho GTPases, and PI3K/Akt, regulating cell adhesion, migration, and gene expression [79,85,86]. RHAMM engagement with low-molecular-weight HA (LMW-HA) activates ERK/MAPK and FAK pathways that promote cell motility and tissue remodeling [87]. Both receptors can create positive feedback loops to *HAS2* expression: more HA production leads to increased receptor signaling, which in turn upregulates *HAS2*, creating a self-amplifying cycle (more *HAS2* → more HA → more CD44/RHAMM signaling → more *HAS2*) [87,88]. However, they engage the feedback loop at opposite ends of the HA molecular weight spectrum in an oscillatory pattern that we argue is central to understanding fascial homeostasis. See Table 5.

HA chain length determines its functional properties and biological activity. HMW-HA, >500 kDa up to several million Da, is synthesized primarily by *HAS2* and sometimes *HAS1*. These long chains create viscous, lubricating, space-filling matrices that maintain tissue hydration and structural integrity. LMW-HA, 10–250 kDa, represents fragmented chains that act as damage-associated molecular patterns (DAMPs), triggering pro-inflammatory and tissue remodeling responses and binding preferentially to RHAMM. Very low-molecular-weight HA (4–25 kDa), generated during acute injury, acts as a potent danger signal, fundamentally switching the behavior of immune cells (especially monocytes/macrophages), stromal cells (fibroblasts), and certain epithelial and tumor cells from homeostasis to active inflammation and tissue remodeling [87,89,90].

This molecular weight-dependent signaling represents a fundamental switch in fibroblasts: HA chain length determines which receptor dominates (CD44 for HMW-HA, RHAMM for LMW-HA), and receptor dominance determines the cellular response (homeostasis versus remodeling). Crucially, both the CD44 and RHAMM pathways couple HA signaling to the calcium-dependent cytoskeletal and ECM remodeling responses [87,91]. We posit that understanding this structure–function relationship is essential for comprehending how fascial tissues coordinate adaptation and maintain homeostasis.

### 6.2. CD44 and RHAMM: Distinct Roles Determined by HA Molecular Weight

#### 6.2.1. HA Chain Length as Signal

To understand how HA signals different cellular responses, we must first understand what HA is at the molecular level: a linear polysaccharide composed of repeating disaccharide units: [GlcA–β1,3–GlcNAc–β1,4]_n_, where GlcA is glucuronic acid and GlcNAc is *N*-acetylglucosamine. HA’s “weight” is a measure of the length of its polymer chain. Research in fibroblasts demonstrates the length of this chain is of conserved significance in the CHA feedback loop.

#### 6.2.2. Low-MW HA and RHAMM: “Attention: Remodel and Strengthen”

Most LMW-HA fragments are generated by the enzymatic or oxidative fragmentation of HMW-HA during normal physiological processes such as tissue remodeling, wound healing, and mechanical stress. As they fragment, their presence becomes increasingly a “danger/change” signal that activates pro-inflammatory pathways and promotes cell migration, proliferation, and tissue remodeling [6,87,92,93,94]. LMW-HA preferentially binds to RHAMM, a receptor that lacks a transmembrane domain but binds HA through a distinct motif. RHAMM functions as a potent regulator of cell motility by activating ERK/MAPK and FAK pathways to drive rapid cell migration, repair, and invasion [87,95,96,97].

RHAMM-mediated calcium signaling is central to CHA axis remodeling, stimulating cytoskeletal reorganization and fibroblast migration, which are essential for tissue adaptation and regeneration. Critically, RHAMM enhances mechanosensitivity by upregulating TRPV4 channel sensitivity, creating a positive feedback loop: LMW-HA activates RHAMM → increased TRPV4 expression → greater Ca^2+^ influx in response to mechanical stimuli → sustained cellular responsiveness to mechanical loading [93]. This Ca^2+^-dependent signaling cascade activates extracellular signal-regulated kinase (ERK1/2) and upregulates matrix metalloprotease-9 (MMP-9) expression in fibroblasts, facilitating ECM remodeling through controlled matrix degradation and enhanced cell motility [90].

#### 6.2.3. High-MW HA and CD44: “Hydrate. Stabilize. Glide. Restore.”

HMW-HA, synthesized during recovery through Ca^2+^-activated *HAS2*, signals homeostasis and safety, maintaining tissue hydration, structural integrity, and anti-inflammatory balance [92,98,99,100,101]. HMW-HA preferentially binds to CD44, which anchors HA to the cell surface and connects it to the actin cytoskeleton, enabling CD44-mediated calcium signaling to regulate cell adhesion, hydration, structural stability, and mechanotransduction by organizing the pericellular HA matrix and cortical actin cytoskeleton [87,93,102]. In contrast to RHAMM’s upregulation of mechanosensitive channels, CD44 downregulates TRPV4 and Piezo1 channels, reducing cellular mechanosensitivity and promoting a quiescent, recovery-oriented state [102].

CD44 also mediates HA uptake and degradation—processes that influence cell proliferation, migration, and cell–cell interactions, which are critical for tissue repair [92,93]. Through regulation of intracellular signaling pathways including RhoA and YAP, CD44 modulates cytoskeletal dynamics and links ECM cues to gene expression and cell cycle progression [66,103,104,105]. CD44 isoforms and their glycosylation variants modulate HA binding affinity and downstream signaling specificity, functioning as a mechanosensitive receptor that integrates biochemical and mechanical signals during the transition from active remodeling to homeostatic stability [102,106]. Overall, the CD44-HMW-HA interaction promotes tissue stability, lubrication, and resolution of inflammation, balancing cellular quiescence and activation to restore safe tissue function and complete the CHA feedback loop [92,98,99,101,102].

## 7. Research Gaps and Experimental Directions for the CHA Axis

### 7.1. Key Translational Gap: Lack of Quantitative Mechanical Thresholds for HA Fragmentation

While the downstream effects of HA fragmentation are well documented, the field lacks quantitative mechanical thresholds that define when physiological loading becomes pathological. See Table 6. HA fragmentation is induced by mechanical overload, oxidative stress, and enzymatic activity (e.g., hyaluronidases, ROS), leading to accumulation of low molecular weight HA (LMW-HA) fragments that act as DAMPs [87,107,108]. These LMW-HA fragments hyper-activate receptors such as RHAMM and CD44, triggering ERK/MAPK signaling, inflammation, myofibroblast differentiation, fibrosis, and neural sensitization—mechanisms.

### 7.2. Addressing the Evidence Gap in Fasciacytes

HA-CD44/RHAMM signaling plays a critical role in regulating progenitor cell migration, proliferation, and tissue remodeling across various mesenchymal cell types, including during embryogenesis and repair. In embryonic myogenesis, HA interactions with CD44 and RHAMM promote myogenic progenitor migration and proliferation, with CD44 mainly influencing proliferation and RHAMM more involved in migration [93]. Similarly, in retinal progenitor cells, HA-CD44 engagement enhances migration, proliferation, and differentiation through signaling pathways involving PKC, Nanog, and PI3K/AKT [115].

RHAMM and CD44 exhibit context-dependent roles in mesenchymal progenitors, where RHAMM acts as a primary HA receptor in non-adherent cells and regulates CD44 expression in adherent cells, indicating a complex interplay that affects HA binding and uptake [116]. Although *HAS2* is established as the main HA synthase in fasciacytes, direct evidence for Ca^2+^-dependent regulation of CD44 and RHAMM in these cells is lacking. Most mechanistic insights come from other cell types like keratinocytes, where extracellular ATP triggers Ca^2+^ influx leading to *HAS2* activation [93]. HA-CD44/RHAMM signaling is broadly implicated in progenitor cell functions relevant to tissue remodeling, demonstrating its importance in fascial adaptation [107,117,118,119]. Specific Ca^2+^-dependent regulatory mechanisms in fasciacytes remain to be demonstrated experimentally.

### 7.3. Promising Experimental Approaches

Current research on HA and *HAS2* in fascia relies heavily on animal and in vitro models, with direct human tissue analysis still limited [10,13,22]. To advance understanding of CD44/RHAMM mechanosensitivity and the Ca^2+^-*HAS2* axis in fasciacytes, the authors propose several promising experimental strategies based on recent advances in mechanobiology and HA receptor studies.

Three-dimensional culture systems offer physiologically relevant platforms for studying fasciacyte mechanotransduction. Culturing fasciacytes in 3D collagen/HA matrices functionalized with HA of varying molecular weights and presentation modes (soluble versus immobilized) can mimic in vivo conditions while enabling precise control of mechanical and biochemical cues. Such systems allow assessment of CD44/RHAMM localization, complex formation, and downstream signaling pathways including ERK1/2, RhoA, and YAP using immunocytochemistry and Förster resonance energy transfer (FRET) microscopy [91,92,120]. Complementary shear stress bioreactors can expose fasciacytes to controlled fluid shear or cyclic stretch, enabling real-time monitoring of receptor expression, localization, and mechanotransduction pathway activation using live imaging and molecular assays [92,121].

Targeted manipulation of CD44 and RHAMM function provides critical tests of receptor necessity and sufficiency. Blocking antibodies or siRNA/shRNA knockdown approaches can inhibit individual receptors, allowing measurement of changes in cell migration, proliferation, Ca^2+^ signaling, and HA synthesis under mechanical stimulation [93,116,122]. Pharmacological modulators targeting downstream effectors—including RhoA inhibitors, YAP regulators, and Ca^2+^ channel blockers—can dissect pathway dependencies and establish causal relationships between CD44/RHAMM activation and specific mechanotransduction outcomes [92,121].

Real-time visualization technologies enable dynamic tracking of CHA loop components in living cells. Ca^2+^-sensitive fluorescent dyes combined with HA-binding fluorescent probes can simultaneously monitor intracellular Ca^2+^ flux and pericellular HA accumulation in fasciacytes within intact fascial tissue or engineered constructs [10,45,121,123]. These approaches parallel successful strategies used in keratinocytes to demonstrate Ca^2+^-dependent *HAS2* activation and can be adapted to fasciacyte-specific contexts.

Single-cell RNA sequencing and proteomic profiling of fasciacytes subjected to mechanical or HA-related stimuli can identify mechanosensitive gene and protein expression signatures unique to fascial tissue, revealing regulatory networks beyond those characterized in other cell types [116]. Integrating these 3D matrix models, live imaging, receptor manipulation, and omics approaches will provide a comprehensive experimental toolkit to directly test CHA loop function in fasciacytes, moving beyond current limitations and enabling physiologically relevant insights into fascial mechanobiology.

### 7.4. Limitations

While the molecular dynamics of calcium and HA synthases (*HAS1/2/3*) are well characterized in other mesenchymal cells, the specific mechanosensitive calcium-HA (CHA) feedback loop in fasciacytes remains unexplored. There is a notable lack of evidence regarding mechanosensitive ion channels, mechanotransductive pathways, and the regulation of HA receptors such as CD44 and RHAMM in fasciacytes, despite their established roles in related cell types [76,124,125,126,127]. This gap highlights the need for targeted research to elucidate the unique signaling mechanisms in fasciacytes and their contribution to fascia health and pathology [10,22,128].

## 8. Conclusions

This review establishes the Ca^2+^-HA (CHA) axis as a unified mechanotransduction feedback loop for fascial adaptation, drawing on extensive evidence from mesenchymal cells and fibroblasts. Fibroblasts play a central role in maintaining fascia’s structural integrity and mechanical responsiveness by producing and remodeling the ECM via collagen and HA extrusion. As such, the authors argue that evidence from this cell type is sufficient for claiming CHA axis application to fascial tissue. The CHA loop integrates mechanical sensing through Ca^2+^ channels, biochemical signaling via HAS activation, and adaptive responses through HA molecular-weight-dependent CD44/RHAMM receptor switching. This “Quiet or Riot” oscillation—between CD44-mediated quiescence during recovery and RHAMM-driven remodeling during stress—provides a mechanistic foundation for understanding how fascial tissues balance homeostasis with adaptive plasticity.

Three key conclusions emerge: First, the CHA framework is well supported by available literature on mesenchymal cells, offering a testable model for fascial mechanobiology. Second, HA molecular weight dynamics and CD44/RHAMM oscillation may have direct implications for optimizing movement, manual therapy, and rehabilitative interventions. Third, while HA-CD44/RHAMM signaling is broadly implicated in tissue remodeling across multiple cell types, Ca^2+^-dependent regulatory mechanisms specific to fasciacytes await experimental demonstration.

A critical translational gap remains: the absence of quantitative mechanical thresholds for HA fragmentation limits our ability to distinguish beneficial from pathological loading. Addressing this gap through systematic in vivo studies, combined with the proposed experimental approaches—3D matrix models, live imaging, receptor manipulation, and omics profiling—may enable translation of the CHA framework from mechanistic insight to clinical practice. Understanding how fascial tissues sense, integrate, and respond to mechanical stimuli through the CHA loop may ultimately transform our approach to movement prescription, manual therapy, and treatment of fascial dysfunction.

## Figures and Tables

**Table 1:** Relationship between mechanical stress parameters and Ca^2+^ entry responses.

Mechanical Stress Parameter	Ca^2+^ Entry Response	Example Systems	Citations
Low intensity, brief	Minimal or transient Ca^2+^ influx	Astrocytes, neurons	[48,49,50]
High intensity, brief	Large, rapid Ca^2+^ spike	Platelets, astrocytes	[49,50,51]
Sustained/repetitive	Prolonged or cumulative Ca^2+^ entry	Endothelial, neurons	[48,49,50,52]

**Table 2:** Mechanosensitive Channel Tuning and Functional Outcomes. How different mechanosensitive channels tune Ca^2+^-*HAS2* responses in fascia.

Channel	Mechanical Stimuli Sensed	Tissue/Cell Specificity	Functional Outcome in Fascia	Citations
Piezo1	Shear, stretch, compression	Widely expressed, force sensors	Initiates Ca^2+^ influx, primary tuning	[55,61,62,63]
TRPV4	Shear, osmotic, moderate stretch	Fluid-exposed, volume-regulating	Sustains/amplifies Ca^2+^, fine-tuning	[55,62]
TRPC5	Stretch, pressure	Select cell types	Additional tuning, context-specific	[64]

**Table 3:** CaMKII—From Ca^2+^ Signal to Lasting Cellular Change. CaMKII’s role in converting Ca^2+^ signals into persistent cellular and tissue changes.

Step	Description/Outcome	Citations
Ca^2+^ binds calmodulin	Activates CaMKII	[71,72,73]
CaMKII autophosphorylation	Maintains activity after Ca^2+^ returns to baseline	[71,72,73,74]
Downstream phosphorylation	Modifies gene expression, cytoskeleton, tissue behavior	[72,73,74]
Structural integration	12-mer holoenzyme, supports elastic network adaptation	[72,73,74]

**Table 4:** Pathological consequences of unchecked *HAS2* activation and excessive HA synthesis.

Pathological State	Mechanism/Consequence	Citations
Edema (Water Retention)	HA’s strong hydrophilic nature leads to water retention and tissue swelling	[80,81]
Impaired Cell Migration	Overly viscous HA-rich matrices hinder cell movement and tissue repair	[77,79,81,82]
Progressive Fibrosis	Excess HA promotes activation of fibroblasts/myofibroblasts, ECM deposition, and scarring	[77,79,83,84]
Tumor Progression	HA-rich stroma supports cancer cell invasion, immune evasion, and metastasis	[16,78,79,82]

**Table 5:** HA molecular weight forms, their primary receptors, and functional signals.

HA Form	Main Receptor	Key Signals/Effects
Low-MW HA	RHAMM	Change, repair, adaptation, remodeling
High-MW HA	CD44	Homeostasis, lubrication, safety, stability

**Table 6:** Evidence for HA fragmentation mechanisms and the critical gap in quantitative mechanical thresholds.

Evidence Area	Findings	Citations
	Well documented across renal, lung, joint, and fascial models	[109,110,111,112,113]
Pathological signaling pathways	LMW-HA triggers RHAMM activation, inflammation, fibrosis, and pain sensitization	[87,89,107,108,112]
Quantitative mechanical thresholds	Not established in any in vivo or clinical studies	[110,114]

## Data Availability

No new data were created or analyzed in this study.
